# Alloy Selection and Manufacturing Technologies for Total Ankle Arthroplasty: A Narrative Review

**DOI:** 10.3390/ma18163770

**Published:** 2025-08-11

**Authors:** Kishen Mitra, Arun K. Movva, Michael O. Sohn, Joshua M. Tennyson, Grayson M. Talaski, Samuel B. Adams, Albert T. Anastasio

**Affiliations:** 1 Department of Orthopaedic Surgery, Duke University School of Medicine, Durham, NC 27710, USA; kishen.mitra@duke.edu (K.M.); samuel.adams@duke.edu (S.B.A.); 2 Feinberg School of Medicine, Northwestern University, Chicago, IL 60611, USA; arun.movva@northwestern.edu (A.K.M.); michael.sohn@northwestern.edu (M.O.S.); joshua.tennyson@northwestern.edu (J.M.T.); 3 Department of Orthopedics and Rehabilitation, University of Iowa, Iowa City, IA 52242, USA; grayson-talaski@uiowa.edu

**Keywords:** total ankle arthroplasty, titanium alloys, cobalt-chromium alloys, beta-titanium, material selection, additive manufacturing, selective laser melting, electron beam melting, biodegradable materials, mechanical properties

## Abstract

Total ankle arthroplasty (TAA) has evolved significantly through advances in alloy selection and manufacturing technologies. This narrative review examines the metallurgical foundations of contemporary TAA implants, analyzing primary alloy systems and their mechanical properties. Cobalt-chromium alloys provide superior mechanical strength and durability but present metal ion release concerns, while titanium alloys (Ti6Al4V) optimize biocompatibility with elastic modulus values (101–113 GPa) closer to bone, despite tribological limitations. Novel β-titanium formulations (Ti-35Nb-7Zr-5Ta, Ti10Mo6Zr4Sn3Nb) eliminate toxic aluminum and vanadium components while achieving lower elastic modulus values (50–85 GPa) that better match cortical bone properties. Manufacturing has transitioned from traditional methods (investment casting, forging, CNC machining) toward additive manufacturing technologies. Selective laser melting and electron beam melting enable patient-specific geometries, controlled porosity, and optimized microstructures, though challenges remain with residual stresses, surface finish requirements, and post-processing needs. Emerging biodegradable materials, composite structures, and hybrid implant designs represent promising future directions for addressing current material limitations. This review provides evidence-based insights for alloy selection and manufacturing approaches, emphasizing the critical role of materials engineering in TAA implant performance and clinical outcomes.

## 1. Introduction

End-stage ankle osteoarthritis (OA) is a common, debilitating condition that can lead to pain, mobility limitation, and decreased quality of life [1,2]. In recent decades, total ankle arthroplasty (TAA) has become a more popular choice among foot and ankle orthopedic surgeons, providing a motion-preserving alternative to arthrodesis [1,3]. However, a proper understanding of TAA material selection and implant design must be considered to ensure acceptable outcomes. Historically, ankle replacement strategies have been in development since the 1970s, when first-generation TAA systems were introduced. These systems were often associated with high failure and complication rates attributed to instrumentation and design flaws, material selection issues, and joint mechanics misunderstandings [2,4]. This often led to complications such as excessive bone-implant interface loading, improper fit, and failure [5].

In order to improve patient outcomes, subsequent generations incorporated superior materials and operative techniques. Mobile-bearing configurations were developed to better emulate the natural anatomy of the ankle and reduce biomechanical mismatch at the implant interface [1,4,6]. Newer systems were distinguished by the utilization of cobalt-chromium and titanium alloys, which provided increased biocompatibility and functionality [1]. Current technologies combine the breakthroughs from previous models and leverage advanced additive technologies to individualize the synthetic ankle joints. These material-driven developments aim to improve device longevity and mechanical reliability [7].

Despite design improvements, TAA implantation is frequently complicated by material and mechanical limitations. The ankle joint’s natural high biomechanical demands (e.g., forces five times higher than body weight when walking) emphasize the need for appropriate materials and precise implantation in order to minimize complications [8]. Other important considerations are metal ion release in cobalt-chromium implants and toxicity associated with the vanadium and aluminum components of titanium-based implants. These toxic effects can manifest both locally and systemically. Local toxicity includes aluminum accumulation in soft tissues and newly formed bone lamella surrounding implants [9]. Systemically, both vanadium and aluminum are associated with serious conditions, including Alzheimer’s disease, peripheral neuropathy, and osteomalacia [10]. In order to mitigate the potential risks of adverse patient outcomes, researchers continue to seek out alternative alloys that can be better tolerated biologically [9,10,11].

The purpose of this review is to establish a deeper understanding of TAA materials science principles, specifically those pertaining to the metallurgical and manufacturing aspects, and their associations with clinical outcomes. This analysis investigates the characteristics and clinical impacts of commonly utilized metal alloys, including cobalt-chromium, titanium, and stainless steel, evaluating their strength, elastic modulus, fatigue resistance, and corrosion behavior. The review compares established manufacturing approaches with innovative additive approaches that can be customized to best accommodate individual patient anatomical differences. The review concludes with an assessment of potential improvements to established TAA protocols. The clinical applicability and potential of novel alloys, biodegradable materials, and composites are considered in the future directions section. The focused synthesis of specific insights of TAA metallurgy and manufacturing elements offers researchers, engineers, clinicians, and patients valuable information in advancing and planning TAA procedures.

## 2. Metallurgical Considerations

Patient goals, biomechanical demands, and biocompatibility must be considered in TAA material selection. The multifactorial requirements necessary to the development of the optimal TAA implant present healthcare personnel with a difficult task. The following section analyzes the principal metallic alloys utilized in TAA applications and their indications. Each alloy’s compositional characteristics, mechanical behavior, and biological performance will be assessed.

### 2.1. Cobalt-Chromium Alloys

Cobalt-chromium (CoCr) alloys are the primary option for most joint replacement procedures, including TAA [12]. The alloy is primarily composed of 58.71–68% cobalt (balance), 27–30% chromium, and 5–7% molybdenum, with trace amounts of other elements [13]. Manufacturing methods can impact mechanical performance, with some studies suggesting that wrought versions (ASTM F1537 [14] and ASTM F799 [15]) can demonstrate increased tensile, fatigue, and corrosion resistance compared to cast versions (ASTM F75 [16]) [17,18].

CoCr provides clinicians with an extremely durable material, which varies based on specific alloy composition. Ultimate tensile strengths can reach 665–2482 MPa, which is comparatively higher than other commonly used materials [19]. The high elastic modulus of CoCr, ranging from 200 to 300 GPa, indicates high durability but can lead to failure due to implant stiffness being mismatched with that of the interfacing bone [19]. The excellent strength and elastic modulus metrics of the material often lead to the attachment of CoCr to the articulating surface of the talus, where its durability is best harnessed in TAA implants [20]. This is often remedied by establishing the interface with a polyethylene or ceramic liner, which promotes smooth gliding [19].

Cytotoxicity, hypersensitivity, and genotoxicity are concerning potential negative effects of CoCr implant integration. While chromium forms a protective oxide layer, passively resisting corrosion, some implants will inevitably undergo corrosion of varying degrees, releasing metal ions and debris [19]. This can cause adverse clinical effects requiring reoperation, including periprosthetic osteolysis and aseptic loosening [19]. Studies suggest that the long-term systemic effects of ion release can lead to debilitating bone necrosis and toxicity [21].

### 2.2. Titanium Alloys

Titanium-based alloys provide a commonly used alternative to CoCr alloys. The Ti6Al4V alloy (ASTM F1472 [22]), consisting of 88.085–91% titanium (balance), 5.5–6.75% aluminum, 3.5–4.5% vanadium, and trace amounts of other elements [23] creates an α + β microstructure configuration that balances strength, corrosion resistance, and biocompatibility to provide a versatile alloy [24].

Titanium alloys, specifically the Ti6Al4V variant, distinguish themselves from other materials due to their low elastic modulus of 101–113 GPa, which is significantly closer to that of cortical bone (5–23 GPa) than comparable materials. The increased biocompatibility paired with an ultimate strength of 860–1173 MPa provides a balanced implant option that can help prevent bone loss from stress shielding, given the relatively lower elastic modulus [24]. Titanium’s primary drawback is its poor tribological properties due to its low resistance to plastic shearing and limited surface oxide protection, limiting its applicability without surface modifications [24].

Titanium implants tend to be well-tolerated following arthroplasty procedures. Once installed, an in vivo oxidation process occurs, providing corrosion resistance, hardness increases, and reduced pitting [25]. These superior osseointegration properties make titanium particularly valuable for tibial components in TAA, where strong bone-implant integration is essential for long-term fixation [20,25].

Similarly to CoCr, toxicity remains a concern in titanium-based alloys. The Ti6Al4V alloy contains vanadium and aluminum, which are associated with Alzheimer’s disease, peripheral neuropathy, and osteomalacia [10]. Titanium-based alloys have repeatedly been shown to raise systemic metal concentrations. In a cohort of 46 patients with Ti6Al4V spinal implants, 34.8% of the patients had abnormal serum titanium and aluminum levels, and 23.9% had abnormal hair metal concentrations at a mean follow-up of five years after surgery [26]. Similarly, an analysis of asymptomatic titanium alloy hip prostheses patients in another study reported 95th percentile vanadium levels of 0.3 µg/L in whole blood, 0.5 µg/L in serum, and 2.8 µg/L in urine, all higher than corresponding values in the unexposed population [27]. These titanium and vanadium metals can accumulate in organs like the liver, spleen, and brain, where high tissue doses can induce neurotoxicity as seen in vitro and in vivo [28]. Other in vitro and in vivo animal studies have also demonstrated clear biological effects at high concentrations. Fibroblast viability drops sharply at vanadium levels of 23 µM, a level close to the 30 µM concentration previously reported in vivo in the peri-implant tissues of patients with poorly functioning Ti6Al4V devices [29]. Another in vitro study suggested that titanium ion concentrations below 15.5 µg/L remain non-cytoxic in vitro [30], and other studies have stratified toxicities by organ to report hepatotoxic titanium concentrations from 30 to 140 µg/L and dermal toxicity at serum titanium levels of 10–30 µg/L [31].

While several studies have quantified these toxic ranges, no professional society has defined formal clinical thresholds for safe versus toxic metal concentrations for Ti6Al4V implants. Unlike in implant-based metal toxicology, environmental metal toxicants and their safety limits are well-established [31]. Establishing acceptable trace-metal limits for implants will require standardization of analytical methods, sampling protocols, reference values, and bioavailability assessments [32]. The potential cellular and systemic effects warrant further study into the long-term effects of titanium implants, while also emphasizing the importance of the development of novel biocompatible materials that mitigate toxicity.

### 2.3. Stainless Steel

Stainless steel has historically been utilized for joint replacement, although its selection has waned with time. The most common alloy (316L, ASTM F138 [33]) is composed of 59.485–64.335% iron (balance), 17–19% chromium, 2.25–3% molybdenum, 13–15% nickel, and trace amounts of other elements [19]. Annealed stainless steel has modest material properties, with a tensile strength of 490 MPa and an elastic modulus of 190 GPa. Steel’s historical utilization and familiarity warrant discussion and consideration, while offering insight into future developments [34].

The chromium components of stainless steel facilitate enhanced mechanical properties and corrosion protection, but corrosion naturally occurs due to anatomical electrochemical responses. This can lead to pitting and is ultimately the cause of 90% of 316L stainless steel implant failures [34]. This phenomenon, combined with the high biomechanical demands of the ankle, has led to the development of alternative variants, such as duplex stainless steel, which can resist these negative consequences [34].

In terms of biocompatibility, stainless steel has been largely replaced by titanium-based alloys. Stainless steel implants are associated with increased fibrous encapsulation than titanium implants, which results in lower biocompatibility [35]. Stainless steel can also cause nickel hypersensitivity reactions, a common adverse effect that must be considered [36]. Stainless steel is best utilized in specific cases where newer alloys are unable to be tolerated, and is no longer primarily utilized for TAA procedures.

As the preceding discussion indicates, there is no clear-cut best option for alloy selection; rather, clinicians and patients must consider individual goals and specific patient anatomical differences. Overall, CoCr provides the highest mechanical strength but raises biological concerns, titanium alloys optimize biocompatibility but have tribological limitations, and stainless steel provides an economical, historically utilized option but lacks high-performance metrics necessary for optimizing patient clinical outcomes. Understanding each alloy’s strengths and weaknesses is critical for selecting the most effective material to restore functionality to patients with end-stage ankle arthritis.

### 2.4. Novel Alloy Formulations

Materials science researchers have made promising advances to address the numerous weaknesses observed in each of the conventionally utilized alloys. β-titanium alloys such as Ti-35Nb-7Zr-5Ta and Ti-29Nb-13Ta-4.6Zr showcase these developments, with the former demonstrating high ultimate tensile strength (630 MPa) and a lower elastic modulus (81 GPa), and the latter exhibiting an excellent elastic modulus (65 GPa) [37,38]. Both of these options present opportunities to enhance biocompatibility when needed.

The Ti10Mo6Zr4Sn3Nb alloy (Ti-B12) presents an excellent, cutting-edge titanium alloy advancement that combines high strength (970 MPa) and optimal elastic modulus (50 GPa). These features, combined with the replacement of potentially toxic components in traditional Ti6Al4V alloys with novel nontoxic materials, lead to strong biocompatibility and safety features [39]. These nontoxic elements, including molybdenum, zirconium, niobium, tantalum, and tin, have been demonstrated to be safe for implantation and exhibit superior biocompatibility compared to aluminum and vanadium-containing alloys [10,40]. Molybdenum and niobium, for example, have been found to be non-toxic at physiological levels and can significantly reduce stress shielding in orthopedic implants [40].

Another novel alloy component, zirconium, presents an opportunity to preserve titanium’s biocompatibility advantages while offering improved durability. This could improve patient outcomes and reduce reoperation rates. Zirconium-based alloys can be varied in their compositions to achieve a range of ultimate tensile strengths and elastic modulus values; aged Zr50Nb50 boasts a 1338 MPa ultimate tensile strength and a 66 GPa elastic modulus value, which shows significant promise for structural and articulating component replacement in TAA [41].

Metal matrix composites that incorporate ceramic materials bring forward another approach to enhance TAA implant strength, wear resistance, and biocompatibility. Bioactive ceramics such as hydroxyapatite, calcium phosphate, and wollastonite foster osseointegration by enhancing bonding [24]. Alumina, zirconia, silica, calcium sulfate, and calcium carbonate offer osteoconductive and osteoinductive properties, but are held back by their biodegradability and brittleness [24]. Composites hold potential utility in combining materials science principles to optimize patient ankle functionality.

**Figure 1:**
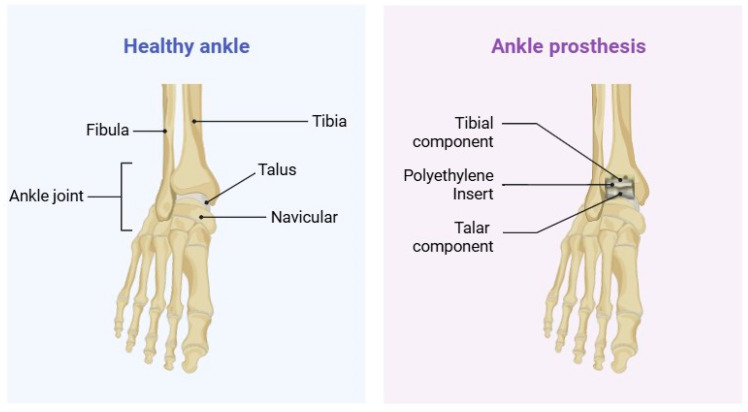
Comparison of healthy ankle anatomy (**left**) and total ankle arthroplasty components replacing the natural joint surfaces (**right**).

Contemporary TAA implant designs strategically combine materials to address specific functional requirements: CoCr alloys for the demanding articulating surfaces of talar components, titanium alloys for tibial components to promote osseointegration, and polyethylene spacers to facilitate smooth joint mechanics (Figure 1) [20].

Table 1 shows a comparative analysis of the primary alloys utilized in TAA procedures. CoCr offers superior durability while titanium alloys promote enhanced biocompatibility. Novel formulations, including β-titanium alloys, can further balance the strengths and weaknesses of the aforementioned alloys and historical materials (e.g., stainless steel).

**Figure 2:**
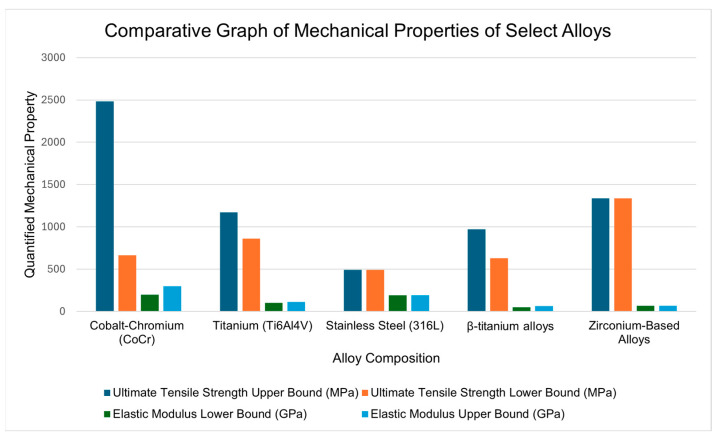
Comparison of mechanical properties of the discussed alloys. Each alloy exhibits superior ultimate tensile strength (high), elastic modulus (low), and corrosion resistance (not quantified) compared to traditional stainless steel, demonstrating the continuing evolution of implant technology.

Figure 2 presents a comparative analysis of the mechanical property ranges for metallic alloys utilized in total ankle arthroplasty. Cobalt-chromium demonstrates the highest ultimate tensile strength values, reaching ~2500 MPa at the upper bound. Zirconium-based alloys achieve high tensile strength (~1400 MPa) while maintaining relatively low elastic modulus values. β-titanium alloys exhibit the lowest elastic modulus range among all materials while sustaining substantial tensile strength (~1000 MPa). Stainless steel shows the most limited mechanical performance across both parameters. The data illustrates that contemporary alloy formulations consistently outperform traditional stainless steel in both strength and modulus characteristics.

### 2.5. Clinical Outcome Analysis

The functional clinical outcomes of three commonly employed alloys, CoCr, Ti6AL4V, and 316L, can be assessed in tandem with their mechanical properties. Cobalt-chrome and titanium-based alloys have been studied for their complication rates in the past decade. Two studies analyzing custom 3D-printed cobalt-chrome implants and 3D-printed titanium implants demonstrated failure rates of 7.9% and 27.8%, respectively [42,43]. The cobalt-chrome implant was associated with a revision rate of 18.4%; so the total reoperation rate for the cobalt-chrome implant was less than the failure rate for the titanium-based implant. Another study suggested that alternative implant formulations, such as those incorporating aluminum or ceramic, produced less polyethylene wear than stainless steel, illustrating a potential consideration in the selection of steel for implant integration [44]. A limitation of these comparisons is a lack of exclusive assessment of implants specific to TAA. Moreover, failures may not be directly attributed to material selection. However, the results of these studies may still provide valuable insights into potential benefits and weaknesses in material selection for foot and ankle surgery applications. Each alloy material provides unique characteristics that can contribute to implant success, and the mechanical properties must be considered alongside clinical indications during each patient’s individualized selection process. The potential of TAA to improve patient clinical outcomes justifies future research into the failure rates and revision rates of various alloys in TAA-specific procedures.

Patient-specific considerations ought to inform the choice of implant material. Namely, in young, active patients, the primary concern is the high mechanical demand that their lifestyle incurs upon the implant. CoCr talar components are well-equipped for this role on account of their high wear-resistance and strength. Conversely, in older, osteoporotic patients, avoidance of periprosthetic fracture becomes the focal point, leading to an increased preference for titanium tibial components on account of their capacity for osseointegration. Medical history might also inform material selection. For example, should a patient possess a hypersensitivity to metals, CoCr and stainless steel alloys ought to be avoided with allergen-free zirconium- and titanium-based alloys taking their place [45]. In these ways, the selection of an implant material is a dynamic one, which must adequately consider the specific biomechanics and medical background of each patient so as to ensure optimal clinical function.

## 3. Manufacturing Methodologies

Producing TAA systems requires the use of advanced manufacturing processes, which deliver on geometric accuracy, fatigue resistance, and mechanical strength. This section discusses the use of both traditional and emerging manufacturing methodologies with regard to TAA system development.

### 3.1. Traditional Manufacturing Techniques

#### 3.1.1. Casting Processes

Investment casting (lost-wax process) is the traditional method by which cobalt-chromium components of TAA implants are manufactured [46]. This process involves pouring ceramic material into a wax pattern to create a mold into which molten metal is ultimately poured [47]. Following the cooling and solidification of this metal, the ceramic cast is removed. While investment casting provides the ability to produce complex geometries with a high degree of accuracy and a desirable surface finish, the manufacturing process can result in the introduction of microstructural carbide precipitates in interdendritic regions, altering the resultant mechanical properties [48]. While these inclusions may result in increased hardness and tensile strength by acting as barriers to dislocation, their presence at grain boundaries can serve as crack initiation sites, leading to a reduction in tensile ductility, which requires careful post-casting treatment for the optimization of implant performance [48,49].

#### 3.1.2. Forging Methodologies

Forging, the formation of implant designs through gross mechanical deformations after heat treatment, is an additional method of TAA system manufacturing, especially for implants containing titanium alloys and cobalt-chromium. While such a process proves more difficult for the construction of intricate geometries than casting and may present with defective microstructures that are difficult to remove upon further heat treatment, it holds the potential to provide improved mechanical properties relative to casting [50,51]. Implants created from forging demonstrate enhanced fatigue strength relative to their casting counterparts, providing an advantage in managing the high-cyclic loading environment of the ankle joint [50,51]. The tradeoff between the ability to manufacture complex geometries and enhanced mechanical properties is one that ought to be considered when selecting a manufacturing method.

#### 3.1.3. CNC Machining

Computer numerical control (CNC) machining, a staple of subtractive machining, has additionally shown merit in the creation of personalized orthopedic implants. Contemporary implants utilize pre-programmed CAM software such as Autodesk Fusion 360 v.2603.1.31 to direct machine tools in machining a desired shape. This machining process is a fast, efficient one that allows for tight tolerances that are necessary in personalized implant design, especially on articulating surfaces [52,53]. As such, this process allows for improved wear characteristics and improved clinical outcomes. Additionally, with the power of modern multi-axis CNC systems, patient-specific implants may be created with even further precision via the utilization of CT data, improving the feasibility of this machining process and allowing for increased personalization based on anatomical differences [54,55]. That said, the machining of titanium alloys presents unique challenges on account of the material’s low thermal conductivity and high chemical reactivity, necessitating specialized machinery and cooling mechanisms so as to best mitigate thermal damage [56].

#### 3.1.4. Heat Treatments and Their Effects on Material Properties

Heat treatment, defined as the controlled heating and cooling of the implant material after its creation, is a key step in the manufacturing of TAA systems. Heat treatment allows for enhanced mechanical properties due to microstructural changes. For cobalt-chromium alloys, heat treatment at temperatures between 1100 °C and 1200 °C allows for the dissolution of carbides and the homogenization of the microstructure, thus improving toughness, hardness, and tensile strength [48,57]. Titanium alloys, on the other hand, undergo mill annealing at slightly lower temperatures, typically between 850 °C and 1050 °C, before enduring controlled cooling to optimize strength-ductility combinations through the control of phase transformations [58,59,60]. Improved fatigue strength through this process can allow for improved long-term clinical performance in TAA systems, but it is imperative to tightly control heat treatment parameters in this step, as minor deviations can result in significant disruptions in microstructure and thus mechanical properties [48,57].

### 3.2. Additive Manufacturing

The shift from traditional manufacturing methods toward additive manufacturing (AM) represents a paradigm change that has revolutionized modern metal manufacturing by enabling the production of highly customized 3D objects where both shape and composition can be tailored to specific requirements [61]. Recent analyses demonstrate that AM offers lower environmental impacts and production costs compared to traditional manufacturing when production volumes are low (approximately 1000 parts per year or less), making it particularly well-suited for patient-specific medical applications [62]. Furthermore, topology optimization techniques commonly employed in AM can reduce material use by 35–65% and energy consumption by 59–91% compared to traditional manufacturing [62].

#### 3.2.1. Electron Beam Melting Processes

Electron beam melting (EBM), the fastest of the AM processes, involves a high-energy electron beam, which is used to melt metal powder layers in a high vacuum environment, allowing for the creation of TAA systems with controlled porosity that mimics trabecular bone structure for the enhanced osseointegration [63,64]. Additionally, the vacuum environment allows for the creation of high-purity systems via the prevention of spontaneous oxidation and accumulation of trace elements upon exposure to air, a problem which is especially prominent in reactive alloys such as titanium [64]. Additionally, this manufacturing process provides impressive geometry control, making it better equipped to personalize implant systems to each patient’s unique anatomy [65]. However, this tight precision can bring about the issue of the trapping of powder within small spaces, a problem which might be solved via a reduced laser point that would decrease productivity and thus increase costs [65]. Additionally, the poor surface finish of systems made by EBM warrants further secondary processes for these implants, further decreasing production speed [66].

Comparative analysis of commercial AM systems demonstrates that EBM operates with significantly thicker layer thicknesses (50–200 μm) compared to SLM (10–100 μm), enabling higher processing speeds while maintaining porosity levels below 1% [67]. EBM produces materials with minimal residual stress due to the high-temperature processing environment and stress-relief characteristics inherent to the vacuum process [67].

#### 3.2.2. Selective Laser Melting Techniques

Selective laser melting (SLM) utilizes a high-powered laser to fully melt metal powder layers, and does so in an inert gas environment as opposed to a vacuum, which allows for the mitigation of oxygen-induced material complications [68]. SLM utilizes a lower processing temperature of 700 °C, which allows for minimization of warping and part distortion, but this comes at the cost of areas of unmelted metal powders in addition to significant residual stresses resulting from large temperature gradients [68,69]. SLM additionally provides the ability to generate impressive geometries with controlled porosities at a lower surface roughness than EBM, but does so at a lower speed [70]. Because of these abilities, SLM is particularly well-equipped for the manufacturing of TAA systems.

SLM achieves superior surface quality with roughness values of 5–15 μm compared to the 20 μm surface roughness observed in EBM processes, though this precision comes at the cost of processing speed [67]. Experimental studies confirm SLM produces significantly smoother surfaces, with average roughness values ranging from 17.4 to 24.4 μm compared to EBM’s 25.6–51.0 μm across different build orientations [70].

Direct metal laser sintering (DMLS) is similar to SLM in that it uses a laser, but does so at lower energy densities so as to partially melt, or sinter, the metal powder instead of fully melting it. As such, compared to the other AM processes, DMLS allows for sharper geometric tolerances and increased porosities relative to traditionally manufactured implant systems [71].

#### 3.2.3. Advantages for Complex Geometries and Patient-Specific Designs

AM technologies provide a design freedom not offered by traditional manufacturing methods of TAA systems, particularly the anatomical optimization of implants to better mirror each patient’s unique geometry. These systems demonstrate increased precision, improved porosity, and by extension, enhanced osseointegration relative to traditionally manufacturing processes, allowing for improved anatomical fit and enhanced stability [64,65]. Furthermore, the use of vacuum environments or inert gases allows for the mitigation of adverse chemical reactions of the implant material, preserving mechanical properties. For these reasons, AM processes are ones that hold the potential to revolutionize TAA system development.

#### 3.2.4. Material Property Considerations Specific to AM Processes

AM processes require unique thermal profiles, which have the potential to significantly influence TAA system mechanical properties via microstructural alterations. The distinctive thermal cycling inherent to these processes creates material characteristics that differ markedly from those achieved through conventional manufacturing methods, necessitating careful consideration of both processing parameters and post-processing strategies.

##### Microstructural Alterations and Thermal Effects

Titanium alloys manufactured via SLM demonstrate increased strength but also exhibit low ductility and significant anisotropy in their mechanical properties on account of acicular α’ martensite microstructures [68,72]. This martensitic microstructure forms due to the rapid cooling rates characteristic of AM processes, creating a hard and brittle non-equilibrium phase that improves tensile strength but is detrimental to ductility [73]. The decomposition of α’ martensite and the subsequent increase in β phase content through post-processing heat treatment reduces the ultimate tensile strength while improving the ductility of the component [73]. As such, post-processing heat treatment is often necessary to decompose this microstructure into the more favorable α + β phase [68].

##### Anisotropy and Build Orientation Effects

The anisotropy in mechanical properties presents an additional challenge in AM-manufactured TAA components, requiring careful alignment of principal stresses in the same direction as the implant’s optimal material orientation [67,74]. Build orientation significantly affects the distribution and magnitude of these anisotropic properties, with implications for both mechanical performance and long-term clinical success [72]. This directional dependence necessitates strategic design considerations to optimize component performance relative to anticipated loading conditions.

##### Powder Characteristics and Processing Variables

The use of powder in these procedures introduces additional variables, with particle size and chemistry serving as important factors to control in the manufacturing process. Powder layers tend to be thicker in EBM than in SLM, contributing to EBM’s increased processing speed, but layer thickness must be carefully balanced against necessary geometric tolerances [63]. Moreover, as has been mentioned previously, a vacuum or inert gas atmosphere is necessary to control for undesirable chemical reactions during processing [64,68]. These atmospheric controls are particularly critical for reactive alloys such as titanium, where exposure to oxygen can significantly compromise material properties.

##### Residual Stress Management

Residual stress management represents a critical challenge in AM processes. SLM-processed Ti-6Al-4V exhibits high residual tensile stresses that can exceed material yield strength, with stress distribution significantly affected by build orientation [75]. The repeated thermal cycling during layer-by-layer deposition creates these elevated stress states, which can lead to component distortion and dimensional inaccuracy if not properly managed. Support structures during SLM processing help minimize residual stresses and prevent component distortion, while build direction optimization is essential for controlling stress gradients [75]. Post-processing techniques, including heat treatment and Hot Isostatic Pressing (HIP), can effectively reduce these residual stresses accumulated during rapid cooling in AM processes [73].

These considerations are material-specific and thus serve as markers for the complexity that comes with shifting away from subtractive manufacturing towards AM in TAA systems. The choice of manufacturing methodology for the creation of TAA systems has a significant impact on the mechanical properties, geometric tolerances, and clinical performance of the resultant implant, with each approach offering distinct advantages and limitations. These are summarized in Table 2.

The transition from subtractive manufacturing processes to additive ones represents a significant change in the focus of TAA production processes, allowing for unprecedented personalization of implants, which can demonstrate enhanced biomechanical sophistication in replicating the natural ankle joint. The precision which these processes provide, when combined with appropriate post-processing strategies, maintains the mechanical properties necessary for clinical success while enabling the potential improvement of TAA performance and patient outcomes as these technologies continue to develop.

### 3.3. Manufacturing in Commercial TAA Products and Clinical Cases

Seven commercial TAA implants have been commonly used in the United States within the last decade, including INBONE II, INFINITY, Salto Talaris, STAR, Trabecular Metal Total Ankle, VANTAGE, and Cadence [76,77]. While most commercial ankles have relied on traditional manufacturing methods, several new and emerging fourth-generation systems have incorporated 3D printing technology, CT-based talus and tibia designs, and patient-specific instrumentation [76,77]. Many of these products incorporate these techniques for pre-operative planning and cutting guides, but products like INFINITY ADAPTIS, Kinos Axiom Total Ankle System, and the APEX 3D Total Ankle Arthroplasty System extend these techniques to implant components to optimize bone ingrowth and to create structure [76,77].

AM is rapidly emerging as a transformative force in TAA by enabling tailored implant designs [78]. Many failures of traditional implants are from prosthesis-to-bone mismatch [46]. AM can address this issue, particularly in ankle replacement with the limited sizes and poor bone stock, as illustrated in one study that produced a full pipeline with additive technology to inform medical imaging, joint modeling, prosthesis design, and 3D printing for ankle replacement [46]. With the rapid fabrication of complex, patient-specific implants and surgical tools and increasing integration of imaging and robotics, AM is poised to advance next-generation orthopedic care for TAA [78].

## 4. Emerging Technologies

The continued progress of materials science serves as a promising new horizon for the development of TAA systems, which might aid in the improvement of clinical outcomes and the mitigation of existing complications. Advanced material systems, including biodegradable scaffolds, hybrid implant configurations, and metal matrix composites, offer innovative solutions to longstanding challenges such as implant loosening, stress shielding, and limited osseointegration. This section further explores active areas of research which hold the potential to further improve the next generation of TAA systems.

### 4.1. Biodegradable Scaffolds and Hybrid Implants

The utilization of biodegradable metallic implants as an alternative to traditional materials is one which holds the capacity to change the future of TAA designs. While permanent implants are designed to serve as the primary source of loadbearing throughout their lifespan, biodegradable materials provide temporary mechanical support while undergoing in vivo degradation and replacement by natural tissue [79,80]. Such a design would eliminate the need for secondary surgeries in addition to mitigating imaging interference on subsequent CT and MRI scans [81]. Additionally, the biocompatibility of these materials might allow for enhanced osteogenesis, improving the initial fixation process [82].

Magnesium-based alloys are one such promising candidate for orthopedic application. Specifically, the release of Mg^2+^ ions from these alloys stimulates the activation of bone morphogenetic proteins (BMPs) and fibroblast growth factor receptors (FGFRs) as well as the differentiation of bone marrow-derived mesenchymal stem cells (BMSCs), allowing for enhanced osseointegration [83]. These alloys could be incorporated into hybrid TAA implants where biodegradable magnesium components enhance osseointegration while permanent, traditional materials provide long-term structural support. While a hybrid design could address some limitations associated with pure magnesium-based implants [84], challenges remain with magnesium’s decreased mechanical strength relative to traditional materials, raising concerns regarding their utility in weight-bearing implants [85]. Additionally, further research is needed into the biosafety of the metabolism intermediates of these implants, especially in patients with renal failure [84]. Nevertheless, their potential to improve fixation and minimize long-term complications warrants continued investigation, especially with regard to TAA systems.

Beyond magnesium, researchers are actively exploring other biodegradable metals, including iron and zinc. Iron demonstrates superior mechanical strength and biocompatibility, marked by stimulation of MAPK and NF-κB pathways that promote bone tissue differentiation by iron ions. Additionally, this material allows for the ability to deliver targeted drugs and growth factors to bone via iron ions, particularly in the presence of a static magnetic field (SMF). However, its slow corrosion rate constrains biodegradability. Conversely, zinc, which is a crucial cofactor for alkaline phosphatase (ALP) and promotes bone differentiation via regulation of macrophage polarization, offers a more favorable degradation rate and good biocompatibility, though it lacks the mechanical strength of conventional alloys [86].

### 4.2. Composite Structures

An additional advanced material of interest for TAA systems is metal matrix composites (MMCs), which consist of a metallic matrix reinforced with dispersed ceramic or other composite particles. Traditional metallic alloy selection often requires balancing the strengths and weaknesses of each material, and these specifications may not be suitable for all patients. Composites address limitations of traditional metallic alloys by optimizing both mechanical and osseointegration properties.

One such example is titanium-ceramic composites, in which the addition of ceramic particles decreases the elastic modulus and hardness of the material, bringing its mechanical properties closer to bone and thus mitigating the effects of stress shielding [87]. As referenced in Section 2.4, ceramic materials such as hydroxyapatite and alumina can be incorporated into alloys to foster bonding [24]. Additionally, the introduction of bioactive composite materials can allow for enhanced fixation and osseointegration [88]. These customizable composites offer a promising path forward in tailoring implant performance and biological compatibility to individual patient profiles, supporting more durable and integrated TAA systems.

TAA systems have significant potential for continued advances in biomaterial selection and integration. Novel developments in biodegradable technologies, including magnesium, iron, and zinc-based alloys, can be incorporated into hybrid implant designs that combine the strengths of multiple materials. Composite structures enhance the osseointegration capabilities of metallic alloys, typically through the strategic incorporation of ceramic materials. Together, these developments provide a promising framework for more durable, individualized, and biologically integrated ankle replacements; these innovations are summarized in Table 3. Continued research and clinical validation of these innovations will be essential to translate their potential into clinical practice.

## 5. Conclusions

TAA developments have been strongly driven by innovations in materials science and manufacturing technologies. Metallurgical and biomechanical advances have played a crucial role in this progress, enabling manufacturers to produce high-performance implants more efficiently. Key considerations include leveraging the durability of cobalt-chromium alloys and the superior osseointegration capabilities of titanium-based materials. Additive manufacturing techniques have revolutionized TAA design by enabling anatomically accurate, patient-specific implants that better match individual anatomy and biomechanical requirements.

While these advancements represent significant progress, opportunities remain for further development of advanced alloy formulations and manufacturing refinements. Novel β-titanium alloys, biodegradable materials, and composite structures offer promising avenues for addressing current limitations in biocompatibility, mechanical properties, and long-term performance; however, the technological readiness of these developments must be considered. Novel β-titanium alloys that prioritize nontoxic material incorporation have not yet achieved the same levels of UTS as traditional titanium; biodegradable materials such as magnesium’s UTS also has not reached that of traditional implants yet, along with potential negative associations with renal toxicity; composite structures, such as those incorporating hydroxyapatite lack sufficient biocompatibility data due to its novelty [24]. While these innovations demonstrate great promise, they are not yet suitable for wide-scale integration. The standardization of additive manufacturing processes and the continued study of long-term biotoxicity of implant materials represent key targets for future implant manufacturing and viability.

The continued evolution of these technologies will likely strengthen TAA’s position as a viable and potentially superior alternative to ankle arthrodesis, providing patients with end-stage ankle arthritis with improved function, mobility, and quality of life.

## Figures and Tables

**Table 1:** Comparison of Metallic Alloys Used in Total Ankle Arthroplasty.

Alloy Type	Composition	Mechanical Properties	Biological Response	Primary Applications
Cobalt-Chromium (CoCr)	Co (58.71–68%), Cr (27–30%), Mo (5–7%), trace elements [13,19]	High strength (UTS 665–2482 MPa) High elastic modulus (200–300 GPa) Excellent wear resistance [19]	Forms a passive oxide layer Potential for metal ion release Cytotoxicity, hypersensitivity, and genotoxicity concerns [19,21]	Articulating surfaces Primary option for most joint replacement procedures [19]
Titanium (Ti6Al4V)	Ti (88.085–91%), Al (5.5–6.75%), V (3.5–4.5%), trace elements [23,24]	Moderate strength (UTS 860–1173 MPa) Low elastic modulus (101–113 GPa) Poor tribological properties [24]	Excellent osseointegration Superior biocompatibility Concerns about Al and V toxicity [10,24,25]	Tibial components Non-articulating surface coatings [24]
Stainless Steel (316L)	Fe (59.485–64.335%), Cr (17–19%), Mo (2.25–3%), Ni (13–15%), trace elements [19]	Moderate strength (UTS 490 MPa) High elastic modulus (190 GPa) Susceptible to pitting corrosion [34]	Acceptable biocompatibility Increased fibrous encapsulation vs. titanium Potential Ni hypersensitivity [35,36]	Limited use in modern TAA systems, Historical utilization [34]
β-titanium alloys	Ti with Nb, Zr, Ta, Mo, Sn (various compositions) [37,38,39]	Ti-35Nb-7Zr-5Ta: UTS 630 MPa, E-modulus 81 GPa Ti-29Nb-13Ta-4.6Zr: E-modulus 65 GPa Ti10Mo6Zr4Sn3Nb: UTS 970 MPa, E-modulus 50 GPa [37,38,39]	Enhanced biocompatibility Improved osseointegration Reduced toxicity concerns by replacing V and Al with non-toxic elements [39]	Emerging applications in newer TAA designs [39]
Zirconium-Based Alloys	Zr with Nb (various compositions) [41]	Aged Zr50Nb50: UTS 1338 MPa, E-modulus 66 GPa [41]	Preserves the biocompatibility advantages of titanium while offering improved durability [41]	Potential for structural and articulating component replacement in TAA [41]

UTS: Ultimate Tensile Strength; E-modulus: Elastic Modulus.

**Table 2:** Comparative Analysis of Manufacturing Methodologies for Total Ankle Arthroplasty Components.

Manufacturing Method	Key Advantages	Limitations	Applications
Investment casting	Complex geometries with high accuracy Desirable surface finish Established process for high volumes [47,48]	Microstructural carbide precipitates in interdendritic regions Potential crack initiation sites at grain boundaries Reduced tensile ductility requiring post-casting treatment [48,49]	CoCr talar components Complex non-articulating surfaces [47]
Forging	Enhanced fatigue strength vs. casting Improved mechanical properties Advantages in high-cyclic loading environments [50,51]	Difficult for intricate geometries Defective microstructures difficult to remove Multiple processing steps [50,51]	High-stress components Tibial baseplates [50]
CNC Machining	Excellent dimensional accuracy Superior surface finish Fast, efficient process with tight tolerances Patient-specific designs using CT data [52,53,54,55]	Material waste Challenges with titanium alloy machining due to low thermal conductivity and high reactivity Requires specialized machinery and cooling [56]	Articulating surfaces Final finishing Patient-specific adjustments [52,54]
Electron Beam Melting (EBM)	Fastest AM process Controlled porosity mimicking trabecular bone High-purity systems via vacuum environment Minimal residual stress [63,64,65,67]	Poor surface finish requiring secondary processing Powder trapping in small spaces Higher energy consumption Reduced productivity [65,66]	Porous fixation surfaces Custom implant geometries Osseointegration enhancement [64,65]
Selective Laser Melting (SLM)	Excellent dimensional precision Fine microstructures Complex geometries with controlled porosity Lower surface roughness than EBM Lower processing temperature minimizes warping [68,70]	Higher residual stresses due to large temperature gradients Areas of unmelted metal powders Need for support structures Lower speed than EBM [68,69,70]	Patient-specific designs Complex geometries Integrated lattice structures [68,70]
Direct Metal Laser Sintering (DMLS)	Controlled porosity gradients Lower energy densities than SLM Sharp geometric tolerances Functionally graded structures [71]	Partial melting/sintering vs. full melting Intermediate density Variable mechanical properties Post-processing requirements [71]	Osseointegration regions Functionally graded components Porous structures [71]

**Table 3:** Emerging Material Technologies for Next-Generation Total Ankle Arthroplasty.

Key Innovation	Description	Potential Benefits	Current Status
Biodegradable Materials	Magnesium, iron, and zinc-based alloys that provide temporary mechanical support with controlled in vivo degradation and replacement by natural tissue	Enhanced osseointegration Reduced imaging interference on CT/MRI scans Improved initial fixation process	Laboratory to preclinical investigation Concerns about mechanical strength in weight-bearing applications Biosafety of metabolism intermediates requires further study [79,80,85]
Hybrid Implant Designs	Engineering approach combining biodegradable components with permanent traditional materials to leverage advantages of both systems	Mitigates drawbacks of standalone biodegradable materials Maintains long-term structural support Allows targeted biodegradation in specific regions Balances temporary support with permanent functionality	Early conceptual and preclinical development Design optimization needed for load distribution Requires further investigation for TAA-specific applications [84]
Metal Matrix Composites (MMCs)	Titanium-ceramic composites with dispersed ceramic particles (e.g., hydroxyapatite, alumina) in a metallic matrix	Decreased elastic modulus and hardness bringing mechanical properties closer to bone Mitigated effects of stress shielding Enhanced fixation and osseointegration through bioactive composite materials Customizable properties to match individual patient profiles	Promising development in TAA material research field Further investigation warranted [87,88]

## Data Availability

No new data were created or analyzed in this study. Data sharing is not applicable to this article.
